# Bibliometric analysis of global migration health research in peer-reviewed literature (2000–2016)

**DOI:** 10.1186/s12889-018-5689-x

**Published:** 2018-06-20

**Authors:** Waleed M. Sweileh, Kolitha Wickramage, Kevin Pottie, Charles Hui, Bayard Roberts, Ansam F. Sawalha, Saed H. Zyoud

**Affiliations:** 1 College of Medicine and Health Science, An-Najah National University, Nablus, Palestine; 2 International Organization for Migration, UN Migration Agency, Migration Health Division, Geneva, Switzerland; 3 Faculty of Medicine, University of Ottawa, Ottawa, CA Canada; 4 London School of Hygiene and Tropical Medicine, London, UK

**Keywords:** Bibliometric analysis, Global migration health, SciVerse Scopus

## Abstract

**Background:**

The health of migrants has become an important issue in global health and foreign policy. Assessing the current status of research activity and identifying gaps in global migration health (GMH) is an important step in mapping the evidence-base and on advocating health needs of migrants and mobile populations. The aim of this study was to analyze globally published peer-reviewed literature in GMH.

**Methods:**

A bibliometric analysis methodology was used. The Scopus database was used to retrieve documents in peer-reviewed journals in GMH for the study period from 2000 to 2016. A group of experts in GMH developed the needed keywords and validated the final search strategy.

**Results:**

The number of retrieved documents was 21,457. Approximately one third (6878; 32.1%) of the retrieved documents were published in the last three years of the study period. In total, 5451 (25.4%) documents were about refugees and asylum seekers, while 1328 (6.2%) were about migrant workers, 440 (2.1%) were about international students, 679 (3.2%) were about victims of human trafficking/smuggling, 26 (0.1%) were about patients’ mobility across international borders, and the remaining documents were about unspecified categories of migrants. The majority of the retrieved documents (10,086; 47.0%) were in psychosocial and mental health domain, while 2945 (13.7%) documents were in infectious diseases, 6819 (31.8%) documents were in health policy and systems, 2759 (12.8%) documents were in maternal and reproductive health, and 1918 (8.9%) were in non-communicable diseases. The contribution of authors and institutions in Asian countries, Latin America, Africa, Middle East, and Eastern European countries was low. Literature in GMH represents the perspectives of high-income migrant destination countries.

**Conclusion:**

Our heat map of research output shows that despite the ever-growing prominence of human mobility across the globe, and Sustainable Development Goals of leaving no one behind, research output on migrants’ health is not consistent with the global migration pattern. A stronger evidence base is needed to enable authorities to make evidence-informed decisions on migration health policy and practice. Research collaboration and networks should be encouraged to prioritize research in GMH.

**Electronic supplementary material:**

The online version of this article (10.1186/s12889-018-5689-x) contains supplementary material, which is available to authorized users.

## Background

The International Organization for Migration (IOM) defines the term migration as “the movement of a person or a group of persons, either across an international border, or within a State” [1]. The term international migration is often used to “refer to movement of people between different countries” while the term international migrant refers to any person who is moving or has moved across an international border away from his/her habitual place of residence for at least one year regardless of the cause, legal status, and length of the stay [1, 2]. The number of international migrants has continued to grow over the past fifteen years reaching 258 million in 2017, up from 248 million in 2015, 222 million in 2010, 191 million in 2005 and 173 million in 2000 [3, 4]. Over 60% of all international migrants live in Asia (80 million) or Europe (78 million). Northern America hosted the third largest number of international migrants (58 million), followed by Africa (25 million), Latin America and the Caribbean (10 million) and Oceania (8 million) [4]. In 2017, two thirds (67%) of all international migrants were living in just twenty countries. The largest number of international migrants (50 million) resided in the United States of America [5]. Saudi Arabia, Germany and the Russian Federation hosted the second, third and fourth largest numbers of international migrants worldwide (around 12 million each), followed by the United Kingdom of Great Britain (UK) and Northern Ireland (nearly 9 million) [4].

The vast majority of international migrants are migrant workers. The term migrant worker refers to all international migrants who are currently employed or are unemployed and seeking employment in their present country of residence [6]. According to recent estimates by International Labour Organization (ILO), there are 150.3 million migrant workers in the world, 55.7% are males and 44.3% are females [6]. Almost half of the migrant workers are concentrated in Northern America and Northern, Southern and Western Europe. The Arab region accounts for 11.7% of all migrant workers.

International migrants who are forced to leave their country of origin due to war or internal conflicts represent another large category of migrants [7]. The global number of forcibly displaced people (FDP) across international borders continues to rise and reached a total of 65 million in 2016. Some of the FDP travel by boat across the Mediterranean Sea seeking a refuge in Europe. According to the United Nation High Commission for Refugees (UNHCR), refugees are people fleeing conflict or persecution [7]. They are defined and protected by international law, and must not be expelled or returned to situations where their life and freedom are at risk [8]. In this regard, a refugee is different from a migrant who chooses to move not because of a direct threat of persecution or death, but mainly to improve their lives by finding work, or in some cases for education, family reunion, or other reasons. Unlike refugees who cannot safely return home, migrants do have the freedom to return home and they will continue to receive the protection of their government [9]. An asylum seeker is defined as is a person who has fled from his or her own country due to fear of persecution and has applied for (legal and physical) protection in another country but has not yet had their claim for protection assessed [10]. By the end of 2016, the total number of refugees and asylum seekers in the world was estimated at 25.9 million representing 10.1% of all international migrants. The developing regions hosted 82.5% of the world’s refugees and asylum seekers [4]. Another category of international migrants is trafficked and smuggled people. Human trafficking differs from smuggling of persons in the use of force and/or deception in order to exploit the victims for sexual business, forced labor or services, slavery or practices similar to slavery, forced begging, or the removal of organs [11]. Although many cases of trafficking in persons do not involve the crossing of international borders, there are some links between cross-border trafficking and regular migration flows [12].

International migration is a multi-phase process that could affect migrants’ life in a positive or negative way [13]. In some cases, international migration can generate benefits for migrants and their families due to wage difference between home country and country of destination [14, 15]. The improved income of the migrant can reflect positively on human development, education and health [16]. For example, according to a recent report by the World Bank, “migrants from the poorest countries, on average, experienced a 15-fold increase in income, a doubling of school enrolment rates, and a 16-fold reduction in child mortality after moving to a developed country” [17]. On the other hand, international migration process carries several risks and costs. Such risks vary according to the stage of the process; pre-departure, travel, destination, interception and return stages [13]. For example, migrants may be subjected to multiple discrimination, violence and exploitation which could affect migrants’ physical and mental health [18–24]. Furthermore, migrants might have difficulties to access appropriate healthcare services or they might face social and language barriers which could affect the quality of health services they receive [25–38]. Some destination countries may not be migrant-sensitive or culturally and linguistically appropriate which negatively affects healthcare services to migrants [24, 39–44]. It is therefore critical for national health systems and policies to address migrants’ right to health, regardless of the legal status of the migrant [24]. Protection of the human rights of migrants, including the rights that address migration-related health vulnerabilities, non-communicable diseases, mental health, occupational health, environmental health, and access to migrant-sensitive health care services must be promoted [45]. Ignoring migrants’ rights to health increases migrants’ vulnerability, creates and amplifies discrimination and health inequalities, incurs higher health costs for migrants and is, in general, a violation of migrants’ rights. On the contrary, delivering equitable access for migrants can reduce health and social costs, improve social cohesion and, most importantly, will protect public health and human rights contributing to healthier migrants in healthier communities [45]. In September 2015, the United Nations (UN) adopted Sustainable Development Goals (SDGs) based on the general principle of “leaving no one behind” [3]. The SDGs set for 2030 called for improving health and human rights of migrants [46].

The thematic working group on research at the 2nd Global Consultation on migration health in 2017 identified the need to “take stock of current research, map the existing landscape of published literature, identify areas of focus and gaps to better organize a global research agenda on migration health” [47]. Quantitative and qualitative analysis of literature in a certain field is called bibliometric analysis in which statistical and mathematical methods are used [48]. Bibliometrics studies in various scientific fields had been published in the past decade [49–52]. Bibliometric analysis differs from systematic reviews, which aim to answer a specific research question based on a limited number of publications [53, 54]. It also differs from scoping reviews, which aim to identify nature and extent of research evidence [55, 56]. Despite these limitations, bibliometric analysis provides an important snapshot of national and international contribution to literature in a particular field. It also provides baseline information, which helps identify research gaps that future studies could focus on [57–61].

Despite the growing numbers of international migrants over the past two decades and increasing global health attention, hitherto, there has not been any assessment on mapping the peer-reviewed literature and examining the global heat map of migration and health literature. Therefore, the aim of this study was to present bibliometric indicators of published literature in global migration health (GMH) pertaining to international migrants. Specifically, the study will examine the growth of publications, authorship, geographical distribution, international research collaboration, important themes discussed, and highly cited articles in the health of international migrants.

## Methods

### Bibliographic database

In bibliometric analysis, documents are retrieved from one single database and analyzed quantitatively and qualitatively [58, 62]. This single database is usually SciVerse Scopus or Web of Knowledge. No grey literature is included in bibliometric analysis. In the current study, SciVerse Scopus, developed by Elsevier, was used to retrieve publications in global migration health (GMH) which refers to global research output on the health of international migrants. Scopus was selected for this study because it has several advantages over other databases such as Web of Science, Medline, and Google Scholar [63–66]. The most important feature of Scopus is its ability to provide bibliometric indicators in a direct and simple way. Furthermore, Medline is 100% included within Scopus and therefore using Scopus will automatically include publications in Medline as well. In the current study, the focus was on documents published in peer-reviewed journals. Therefore, grey literature, conference proceedings, and books/book chapters were not included in the analysis. The study period of the current study was limited from year 2000 to 2016.

### Search strategy

#### Inclusion step

The strategy developed was based on constructing a separate search strategy for each component in the spectrum of the definition of international migrants. Therefore, a search query was developed for the following components: (1) migrant workers, (2) refugees/asylum seekers/displaced people (not internally displaced), (3) international students, (4) trafficked victims/victims of human smuggling, (5) patients’ mobility across borders, and (6) international migrants/immigration. The search queries number 1 to 6 were connected with “OR” operator. The keywords used in each component were partially obtained from published systematic reviews [5, 10, 25–31]. Furthermore, international experts in the field of GMH were consulted and reviewed the keywords. The outcome of these search queries was combined with the “health” component to produce health-related migration literature. The health component of the study consisted of more than 70 keywords in health and in social determinants of health entered in title-abstract-key. For example, any document in which one of the following words is present in title or abstract or key were considered health-related literature: (health* or medicine or poverty or discrimination or disease, or trauma or emergency or inequal* or infect* or “quality of life” or mental or clinic*) (Additional file 1). Some of these keywords were from certain systematic reviews and some were extracted from mesh search for the word “health” in Medline [5, 67–75]. In addition to this, these health keywords were also confined to documents that are indexed in Scopus under the subject areas of medicine, nursing, social, humanities, psychology, pharmacology, microbiology, neuroscience, dentistry, biochemistry/biology, and health professions. In the search query, asterisk and the quotation marks were used to increase the efficiency of the search strategy.

#### Exclusion step

An exclusion step was added to the search strategy. The purpose of this exclusion step was to eliminate all potential false positive results. The following criteria were implemented in the exclusion step:

1. The duration of the study was set from 2000 to 2016 and all other years were excluded. No language restriction was imposed.
2. Scopus has a function which divides the retrieved documents into various subject areas based on the field and scope of the publishing journal. All documents published in journals indexed in the subject areas of energy, chemical engineering, material science, mathematics, physics, computer science, and geology and earth studies were excluded after confirming that the retrieved documents in these subject areas were irrelevant to GMH.
3. Documents with certain keywords, regardless of the subject area of the document, were also excluded since these documents are related to internal migration or to topics in the field of internal migration, botany or cell biology or genetics or industry or veterinary.
4. Documents pertaining to brain drain and migrant nurses or physicians or health professions were excluded.
5. Documents published in certain specific journals were excluded because they were irrelevant to GMH (e.g. *Journal of Mammalogy* which includes documents about migration of animals).

Two co-authors (S.Z and A.S) were responsible for finding false positive results based on the above-stated criteria after consultation with a third author (W.S). The search for false positive results continued for three months and was carried out by manually reading through the retrieved documents.

#### The validity of the search strategy

In the current study, false positive results were minimized by using the title search. The use of title/abstract search is known to retrieve many false positive results. Furthermore, some of the keywords used in this study might be also used in other scientific disciplines such as molecular biology, genetics, botany, and veterinary. Therefore, some of the authors had to search for false positive results. The absence of false positive results was confirmed by (1) testing the top 200 cited articled for the absence of false positive documents, and (2) testing all subject areas and journal names that had a minimum of two published documents. Therefore, in the worst-case scenario, the false positive results will not exceed 100 documents if we assume that that the remaining untested journals and subject areas included false positive results. This means that the maximum potential percentage of false positive results did not exceed 0.5% of the total results.

#### The absence of false negative results

To test for the absence of false negative results, we compared two different methods of data collection. In the first one, we collected data regarding research output (number of publications) for each of the most active authors as obtained through the search strategy, whilst in the second one, the research output of each of the most active authors was extracted and reviewed by exploring the author profile as presented by Scopus. The extent of agreement between the two methods is measured by interclass correlation coefficient using SPSS [76–80]. An excellent agreement between the two methods with an interclass correlation above 95% and a *p*-value less than 5% is indicative of a high validity of the search strategy. In the current study, the interclass correlation was 0.98% and the p-value was 0.003. The authors also contacted one of the active authors mentioned in the list to confirm that the results we obtained matched his research output which he positively confirmed.

#### Data analysis and visualization

Retrieved data were exported from Scopus to Excel for analysis and tabulated. The data exported included

1. Number of publications for each country,
2. Names of journals in which the retrieved documents appeared along with the number of documents published by each journal,
3. Names of authors with their Scopus affiliation and number of publications for each author,
4. Names of institutions/organizations with the number of publications for each institution,
5. The top cited articles with their referencing details,
6. The types of documents with the frequency of each type,
7. The title, authors, and journal details of all retrieved documents to be used for VOSviewer mapping, and
8. The number of publications in each year of the study period

For authorship analysis, data were initially sorted based on the number of authors. Then, single-authored documents were separated from multi-authored publications and the percentage of each type was calculated. Retrieved data were also sorted based on the number of different country affiliations per article to calculate international collaboration. Documents with authors from different countries are called multiple country publications (MCP) which represent international or inter-country collaboration. On the author hand, documents in which all authors have one country affiliation are called single country publication (SCP) and represent intra-country collaboration. The percentage of MCP reflects the extent of international collaboration for each country. Research interests of most active authors were obtained from personal sites of the active authors such as ResearchGate, LinkedIn, university web site, Google Scholar, and others.

#### Research domains

The retrieved data were also analyzed for the volume of each of the following five research domain: (1) psychosocial and mental health; (2) infectious diseases; (3) non-communicable diseases (NCDs); (4) maternal and reproductive health; and (5) health policy and systems. The volume of each research domain was determined by limiting the retrieved documents to title keywords relevant to each research domain. These limiting keywords were developed by the authors themselves. In the current study, the domain of health policy and systems was defined as documents on the health system functions of regulation, organization, financing and delivery of services, as well as broader determinants such as social and economic policies directly affecting the health system. Examples of keywords used to retrieve documents in health policy and systems domain included health system, health services, access to healthcare, human rights, rights to health, hospitalization, medical services, emergency room visits, and migration policies [81]. For maternal and reproductive health domain, examples of keywords used included pregnancy, reproductive health, maternal health, newborn, and gynecology.

In this study, Hirsch-index (*h*-index) was used as a measure of the impact of publications. Hirsch-index is defined as the number of articles (n) that have received at least n citations [57]. Figures were created using Statistical Package for Social Sciences (SPSS). VOSviewer software was used to create visualization maps while ArcMap 10.1 was used to create the geographical distribution of the retrieved documents [82–84]. For VOSviewer mapping of most frequent author keywords, a minimum occurrence of 65 was used as a cutoff point for inclusion of the keyword in mapping analysis. The analysis also included distribution of publications based on income as defined by World Bank country classification [85].

#### Ethical consideration

The study did not include human subjects or human materials and ethical approval of the study was not required based on guidance from the institutional review board of An-Najah National University.

## Results

### Number and types of retrieved documents

**Fig. 1:**
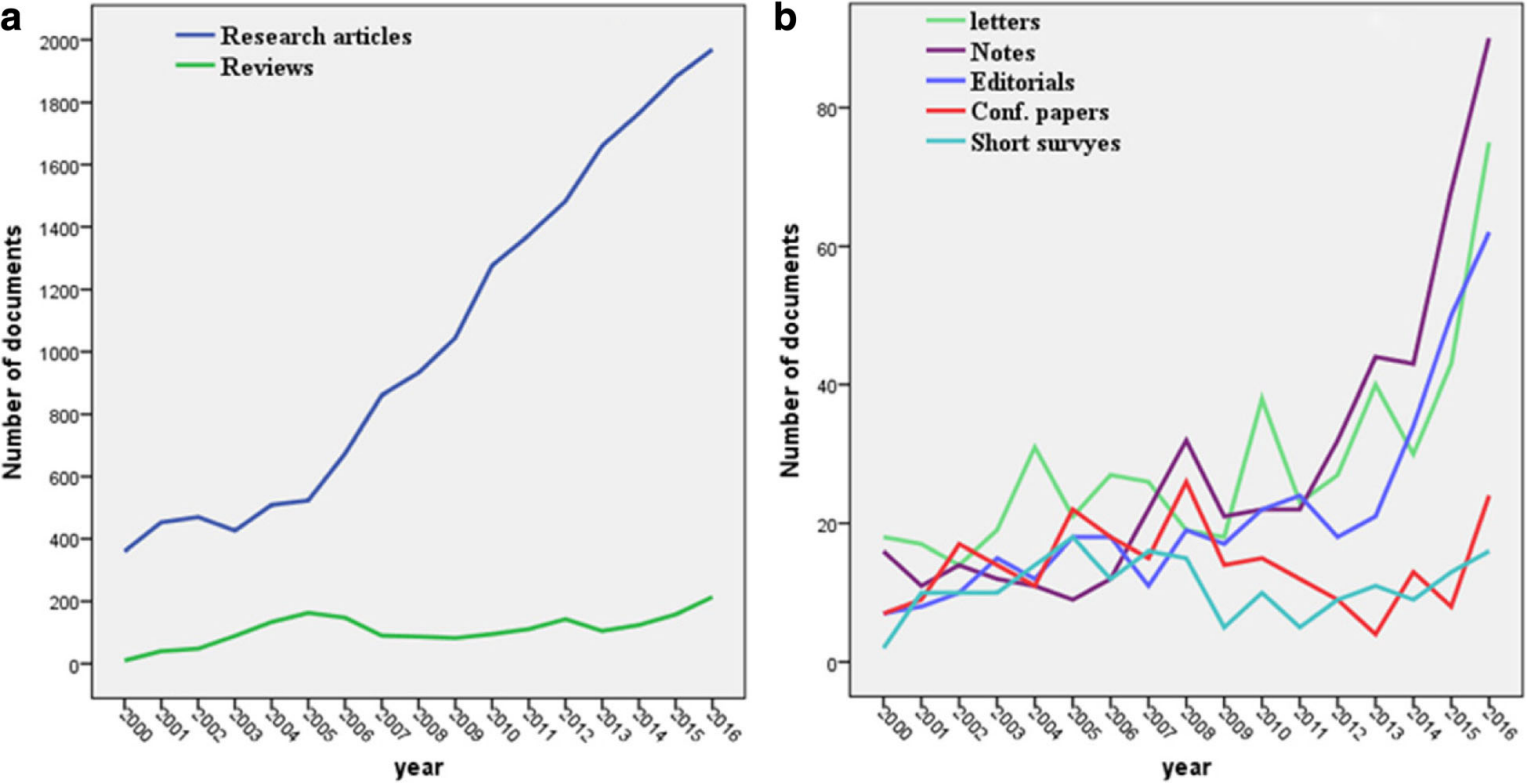
Annual growth of publications in research articles, reviews (**a**), letters, notes, editorials, conference papers, and short surveys (**b**)

The numbers of retrieved documents in each step in the research strategy are shown in the supplement (Additional file 2). The search strategy retrieved a net total of 21,457 documents. The majority were research articles (17,606; 82.1%) followed by review articles (1835; 8.6%), letters (488; 2.3%), notes (487; 2.3%), editorials (367; 1.7%), conference papers (238; 1.1%), short surveys (186; 0.9%) and articles in press (250; 1.1%). Figure 1a and b show the annual growth of different types of retrieved documents. English (18,977; 88.4%) was the dominant language followed by Spanish (789; 3.7%), German (674; 3.1%), French (456; 2.1%), and Italian (205; 1.0%). During the study period, there was a linear growth of the number of publications (R^2^ = 0.98). The maximum annual number of published documents was 2578 (12.0%). Approximately, one third (6878; 32.1%) of the retrieved documents were published in the last three years of the study period (2014–2016).

### Visualization of author keywords

**Fig. 2:**
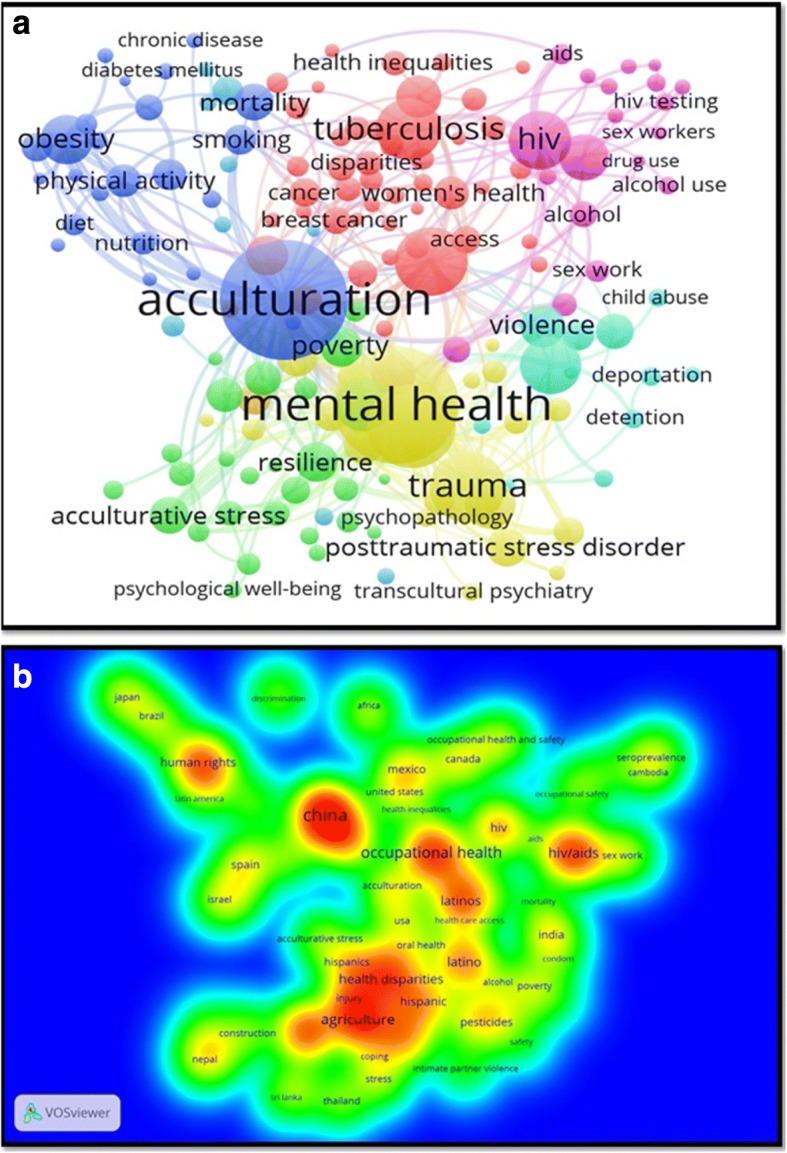
(**a**) Network visualization map of author keywords in GMH. (**b**) Density visualization map of author keywords in literature pertaining to migrant workers

The visualization map of author keywords showed that “mental health”, acculturation, depression/suicide, HIV/AIDS, health disparities/equities/inequalities, discrimination/racism/prejudice, trauma/PTSD, violence, and substance use/abuse were the most frequently encountered author keywords (Fig. 2a).

### Research domains

Further analysis showed that the vast majority of the retrieved documents (10,086; 47.0%) were on psychosocial and mental health. The retrieved literature in GMH also included 6819 (31.8%) documents in health policy and systems, 2945 (13.7%) documents in infectious diseases, 2759 (12.8%) documents on maternal and reproductive health, and 1918 (8.9%) on NCDs. Due to overlap among various domains, the total percentages of research domains exceeded 100%.

### Typology of migrants

Based on title search, 5451 (25.4%) documents were about refugees/asylum seekers, 1328 (6.2%) were about migrant workers, 440 (2.1%) were about international students, 679 (3.2%) were about human trafficking/smuggling, and 26 (0.1%) were about patients’ mobility across international borders. However, 13,533 (63.1%) documents did not include any specific type of international migrants in their title. Of the total 1328 documents about migrant workers, 292 (22.0%) documents were about Latin American and Mexican workers, 226 (17.0%) documents were about Hispanics, 564 (42.5%) documents were about Asian workers, and 81 (6.0%) documents were about African migrant workers. The remaining documents did not include ethnicity of migrant workers in the document title/abstract. Figure 2b is a density visualization map of most frequent author keywords in literature pertaining to migrant workers.

### Preferred journals

The retrieved documents were published in 4228 different peer-reviewed journals. The top ten preferred journals for publications in the field of GMH was *Journal of Immigrant and Minority Health* (747; 3.5%) followed by *BMC Public Health* (229, 1.1%) (Table 1). The list included five journals in the field of migration/refugee while the remaining journals were in the field of public health, social medicine, culture, or general medicine. Publications in *Social Science and Medicine* journal received the highest citations per documents (37.1 citations/document) followed by those published in *American Journal of Public Health* (36.1 citations per document).

**Table 1:** List of top ten active journals with in GMH (2000–2016)

Journal	Frequency *N* = 21,457	%	C	C/A
*Journal of Immigrant and Minority Health*	747	3.5	5560	7.4
*BMC Public Health*	229	1.1	2919	12.7
*Social Science and Medicine*	225	1.0	8341	37.1
*International Migration*	205	1.0	2247	11.0
*International Journal of Migration Health and Social Care*	198	0.9	314	1.6
*Journal of Immigrant and Refugee Studies*	175	0.8	592	3.4
*Journal of Refugee Studies*	172	0.8	2732	15.9
*Lancet*	163	0.8	2496	15.3
*Plos One*	161	0.8	1280	8.0
*American Journal of Public Health*	147	0.7	5307	36.1

*GMH* = global migration health; *C* = citations; *C/A* = citations per article

### Most active countries

**Fig. 3:**
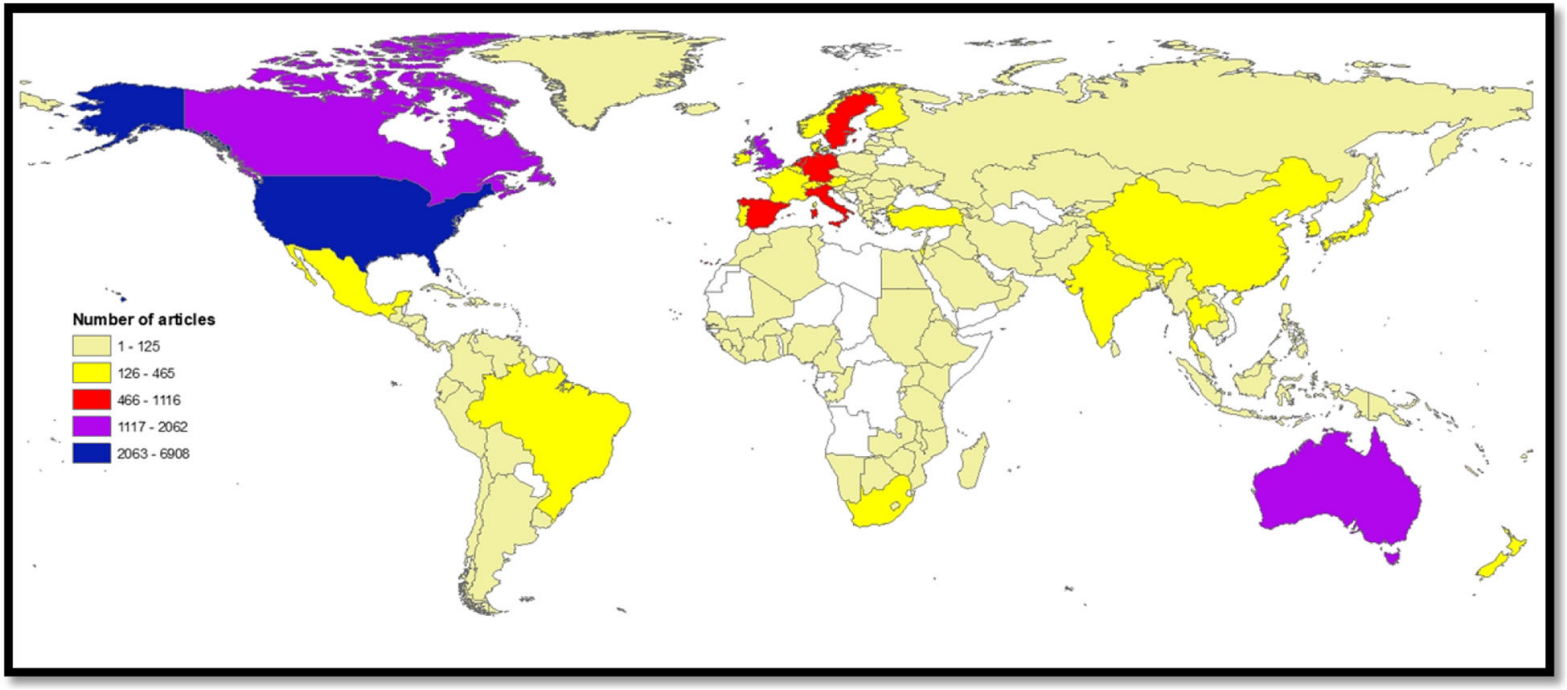
Geographical distribution of retrieved documents in GMH (2000–2016). Areas with no color in the map represent regions with no data available or no research output in the field of GMH

**Fig. 4:**
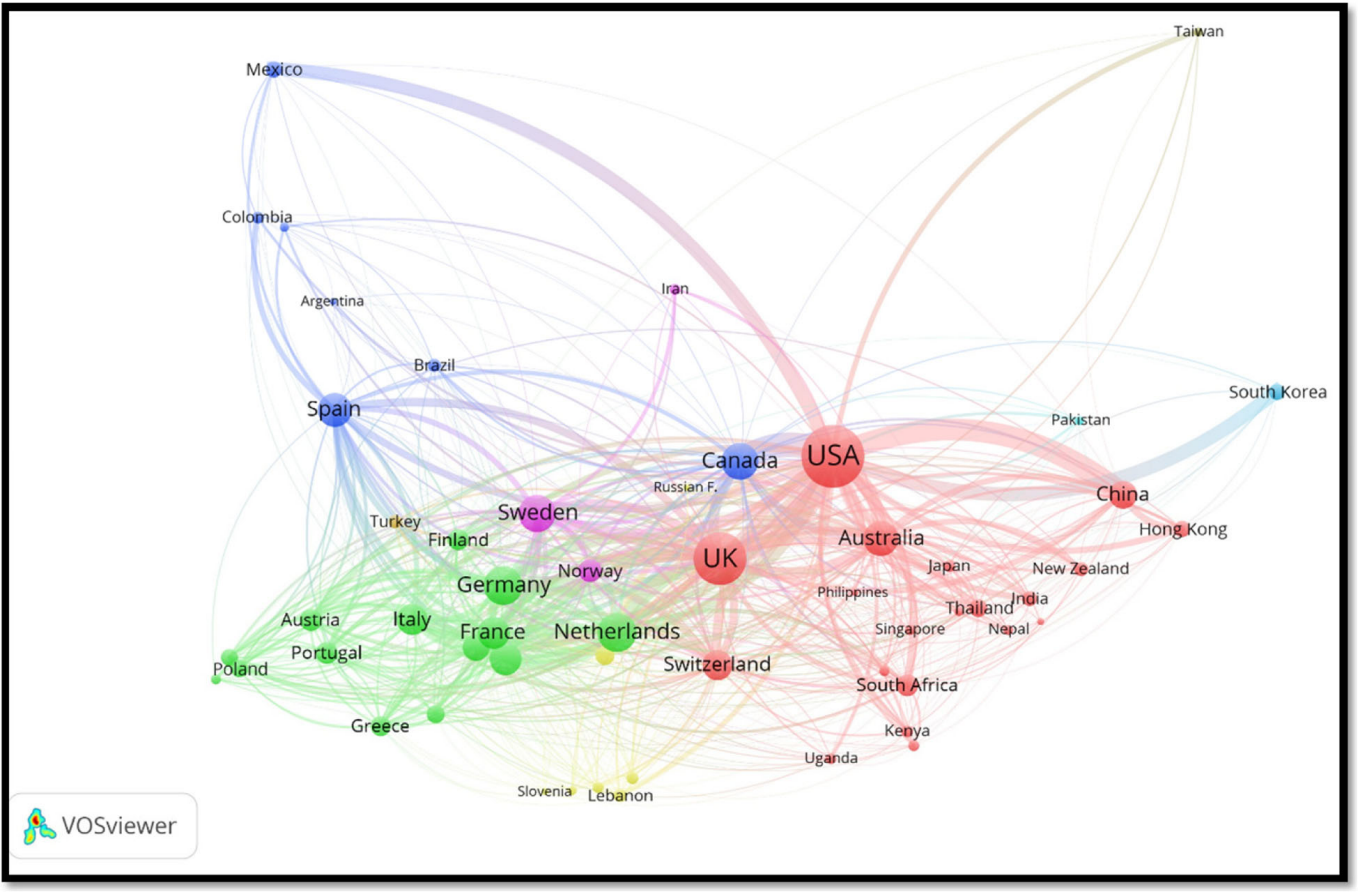
International collaboration in GMH among countries with a minimum productivity of 10 documents

Researchers from 156 different countries participated in the retrieved documents. The ten countries with the highest research output in GMH are shown in Table 2 while the geographical distribution of retrieved documents is shown in Fig. 3. The USA ranked first (6908; 32.2%) followed by the UK (2062; 9.6%). Publications from the Netherlands had the highest number of citations per documents followed by those from the USA and Sweden. In the top ten active countries, Sweden had the highest percentage of documents with international collaboration followed by Netherlands and UK. Overall, the range of international collaboration was as high as 40.5% for Sweden and as low as 21.6% for the USA. It must be noted here that the USA had the lowest international collaboration as a percentage of its total research productivity. However, this low percentage represents a large number of documents with international collaboration when compared with that from Sweden despite that Sweden has a high percentage of international collaboration. International collaboration among countries with a minimum productivity of 20 documents in the field of GMH is shown in Fig. 4. In the map, the thickness of the line connecting between countries represents the strength of research collaboration.

**Table 2:** List of top ten active countries and extent of research collaboration in GMH (2000–2016)

Country	Frequency	% N = 21,457	C/A	Intra-country collaboration	%	Inter – country collaboration^a^	%
USA	6908	32.2	14.7	5417	78.4	1491	21.6
UK	2062	9.6	13.4	1312	63.6	750	36.4
Canada	1648	7.7	13.7	1208	73.3	440	26.7
Australia	1401	6.5	11.7	1048	74.8	353	25.2
Germany	1116	5.2	8.8	799	71.6	317	28.4
Spain	1017	4.7	6.3	779	76.6	238	23.4
Netherlands	845	3.9	15.9	524	62.0	321	38.0
Sweden	693	3.2	14.1	412	59.5	281	40.5
Italy	604	2.8	7.5	454	75.2	150	24.8
France	465	2.2	8.5	302	64.9	163	35.1

^a^Inter-country collaboration = International collaboration; C/A = citations per article

### Most active institutions

The ten most productive institutions/organizations are shown in Table 3. The *University of Toronto* ranked first with 525 (2.4%) publications followed by *University of Amsterdam* (325; 1.5%) and *Columbia University in the City of New York* (301; 1.4%). The top ten active institutions/organizations included three in the USA, two in Australia, two in Canada, one in the UK, one in Sweden, and one in the Netherlands. Analysis of the retrieved documents showed that 182 (0.8%) documents included authors with affiliation pertaining to UN, International Organization for Migration (IOM), the World Health Organization (WHO), or international organization.

**Table 3:** List of top ten active institutions/organizations in GMH (2000–2016)

Institutions/organizations	Number	% N = 21,457	Country
*University of Toronto*	525	2.4	Canada
*University of Amsterdam*	325	1.5	Netherlands
*Columbia University in the City of New York*	301	1.4	USA
*Karolinska Institutet*	265	1.2	Sweden
*University of California, Los Angeles*	228	1.1	USA
*McGill University*	191	0.9	Canada
*Centers for Disease Control and Prevention*	190	0.9	USA
*University of Oxford*	188	0.9	UK
*University of New South Wales UNSW Australia*	182	0.8	Australia
*University of Melbourne*	179	0.8	Australia

Analysis of research output based on income showed that low-income countries contributed to 162 documents (0.8%). High-income countries contributed to 19,220 (89.6) while middle-income countries, both lower and upper-middle income, contributed to 2386 (11.1%) taking into consideration the potential overlap in research output among countries with different economies. Analysis of retrieved literature based on WHO regions showed that the region of Americas had the highest research contribution (47.1%) followed by the European region (42.7%). The Eastern Mediterranean region (1.8%) had the least contribution.

### Most cited articles

The top ten cited articles are shown in Table 4. The article that received the highest citation was a review article published in Lancet in 2005 and discussed the prevalence of mental disorders in a large number of refugees in Western countries [86]. In line with earlier stated results, the majority of top ten cited articles were in psychiatry/psychology. None of the top ten cited articles was about infectious disease and none was about migrant workers in specific.

**Table 4:** Top ten cited documents in GMH (2000–2016)

Title	Reference	Journal	Cited by
“Prevalence of serious mental disorder in 7000 refugees resettled in western countries: A systematic review”	[86]	*Lancet*	574
“Ethnic identity, immigration, and well-being: An interactional perspective”	[156]	*Journal of Social Issues*	534
“A psychology of immigration”	[157]	*Journal of Social Issues*	504
“Rethinking the concept of acculturation: Implications for theory and research”	[158]	*American Psychologist*	440
“Immigrant youth: Acculturation, identity, and adaptation”	[159]	*Applied Psychology*	440
“Ethnic-immigrant differentials in health behaviors, morbidity, and cause-specific mortality in the United States: An analysis of two national data bases”	[136]	*Human Biology*	414
“Migration and mental health”	[96]	*Acta Psychiatrica Scandinavica*	394
“Insights into the ‘healthy immigrant effect’: Health status and health service use of immigrants to Canada”	[160]	*Social Science and Medicine*	381
“Acculturation and overweight-related behaviors among Hispanic immigrants to the US: The National Longitudinal Study of Adolescent Health”	[161]	*Social Science and Medicine*	378
“Prevalence of mental illness in immigrant and non-immigrant U.S. Latino groups”	[162]	*American Journal of Psychiatry*	371

### Authorship analysis

The total number of authors participated in publishing retrieved documents was 66,295, a mean of 3.1 authors per document. Single–authored publications constituted approximately 25% of retrieved documents while the remaining were multi-authored (≥2 author publications). The top ten active authors in the field of GMH are shown in Table 5. The top ten list included three from the USA, two from Australia, one from Canada, two from Denmark, one from the Netherlands, one from Germany, and from Sweden. Research collaboration and networking of the most active authors showed that active authors exist in six separate clusters (data not shown). Some of the active authors exit together in one cluster. For example, (1) Steel, Z. and Silove, D. exist in one research cluster; (2) Arcury, T.A; Quandt, S.A exist in one cluster; (3) Krasnik, A.; Razum, O.; Norredam, M exist in one cluster.

**Table 5:** List of top ten active authors in the field of GMH (2000–2016)

Rank	Author	Frequency	% N = 21,457	C/A	Country	Research interest
1st	Arcury, T.A.	86	0.40	18.6	USA	Agriculture; Transients and Migrants; Hispanic Americans; North Carolina; Agricultural Workers’ Diseases
2nd	Quandt, S.A.	79	0.37	19.2	USA	Hispanic Americans; Agriculture; Transients and Migrants; North Carolina; Occupational Diseases
3rd	Razum, O.	72	0.34	14.5	Denmark	Health services research among different social and ethnic groups.
4th	Norredam, M.	61	0.28	12.6	Germany	Migration, social inequality in health, health system research, screening, vaccination, global health
5th	Krasnik, A.	55	0.26	16.8	Denmark	Equity and health; migration and health and health services research. A particular focus is on the impact of ethnicity and migration on health conditions and access to health care; vulnerable migrant groups; mental health and chronic diseases among migrants.
6th	Silove, D.	54	0.25	36.8	Australia	Refugee and post-conflict mental health trauma psychiatry separation, anxiety, and human rights
7th	Hinton, D. E	46	0.21	32.9	USA	Medical Anthropology, Health Psychology, Abnormal Psychology, mental illness, Psychopathology
8th	Sundquist, J.	44	0.21	23.4	Sweden	family and community medicine and public health, health of racial/ethnic minority groups, immigrants and refugees, influence of social and familial environments on psychiatric and drug use disorders
9th	Rousseau, C.	43	0.20	20.6	Canada	Transcultural Child Psychiatry, refugee children, torture victims. Refugee children and adolescents from Southeast Asia, Central America, and Somalia.
10th	Renzaho, A.M.N.	38	0.18	13.5	Australia	Public Health and Nutritional Epidemiology. Migration and health, complex humanitarian emergencies, and development aid.
10th	Steel, Z.	38	0.18	53.0	Australia	Trauma and mental health, psychological intervention in complex emergencies and management of traumatic disorders, cross cultural differences in psychiatric epidemiology, refugee mental health, mental health and human rights.
10th	Stronks, K	38	0.18	17.1	Netherlands	Influence of the social context on health and illness.

### Visualization of author keywords on the mental health domain

**Fig. 5:**
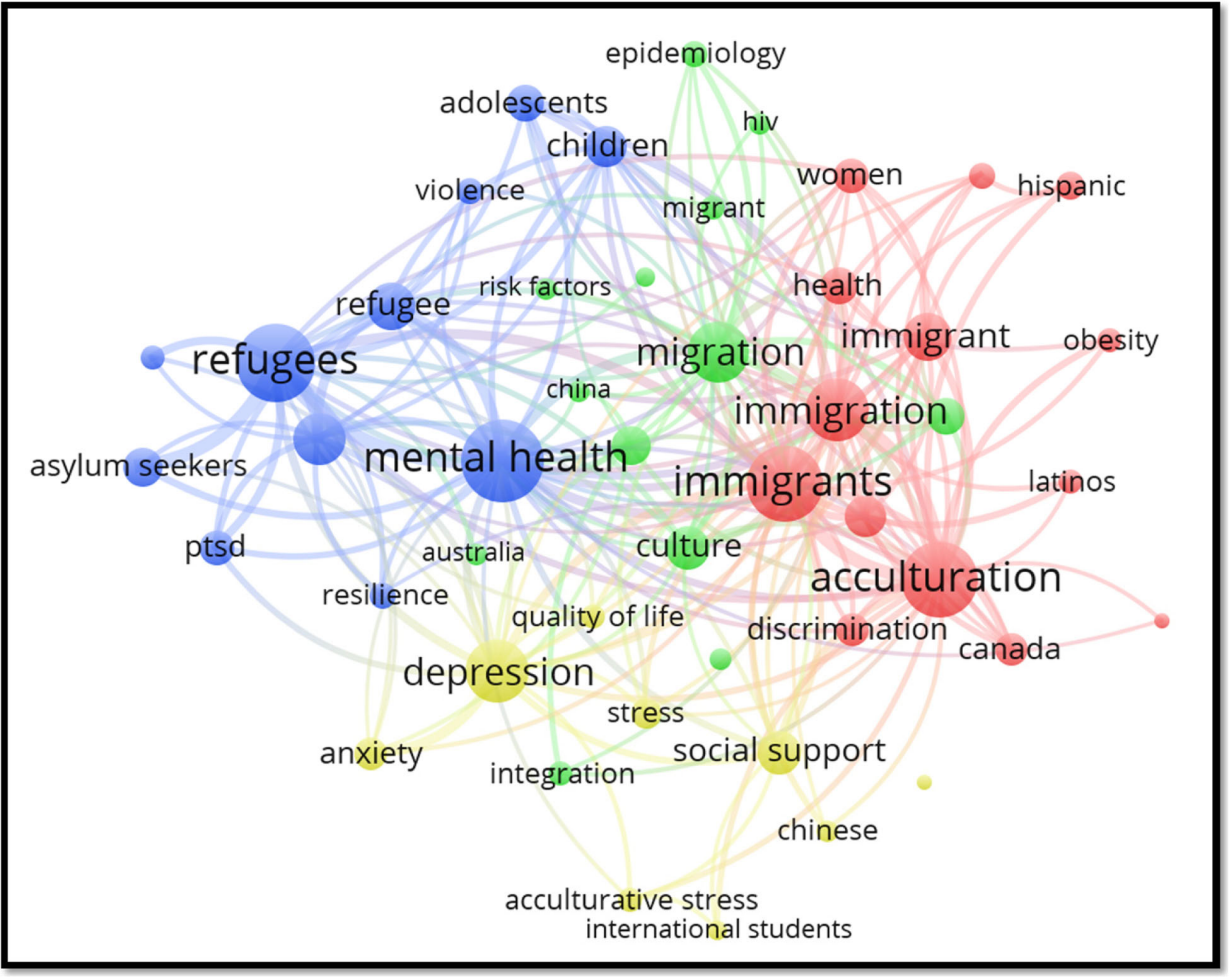
Most frequent author keywords in documents pertaining to mental health in GMH

Since mental health dominated the GMH literature, we further analyzed and visualized author keywords of documents pertaining to mental health. The visualized map showed that there were four clusters of frequent keywords (Fig. 5). Keywords such as mental health and post-traumatic stress syndrome were associated with keywords related to refugees and asylum seekers (blue cluster). Keywords such as acculturation and Latinos were associated with immigration-related keywords (red cluster). The third cluster (yellowish green) included keywords such as depression, anxiety, and stress. These keywords were almost evenly spaced between the cluster of refugees and that for immigrants. The third cluster also included a keyword pertaining to international students. The fourth cluster [87] included keywords such as culture, integration, HIV, and migrants.

## Discussion

### Growth of publications

The results of this study showed that there was a linear upward increase in the number of publications, which is in parallel to the global increase in numbers of migrants and most likely the number of migrant health researchers. Several bibliometric studies from different scientific fields also showed an increase in the number of publications, suggesting the growth of annual publications in GMH also reflects an overall growth of global scientific research [88–91]. The linear increase in the number of publications in GMH might reflect the increase in operational activities of international organizations in meeting health needs of migrants and disadvantaged people across the globe. In the past several years, government officials, researchers, and human right activists in host countries were involved in investigating the health status and needs of refugees and migrants, not only to provide humanitarian aid but also to limit the potential spread of certain infectious diseases among migrants and refugees [92–95].

### Domains of GMH research

Research within the psychosocial and mental health domain yielded the greatest research output within the GMH literature. This is understandable given the impact of war and disaster-related trauma and the subsequent journey of migration which can manifest later as mental stressors arising from acculturation [86, 96–99]. Mental health is one of the leading cause of disability life-years particularly in low and middle-income countries [100], which emphasize the importance of mental health within GMH research. Results of the current study indicated that approximately 14% of GMH literature was in the field of infectious diseases, and most studies were concentrated in high-income countries with immigration and refugee resettlement programs. The risk of infectious diseases in countries of origin of international migrants is higher than that in destination countries [101]. Globalization of infectious disease through travel and migration remain a priority to address challenges of cross-border disease control, ensure global health security and prevention of new, emerging and reintroduction of infectious diseases through human mobility corridors. Migrants and refugees arriving from regions of the world with different epidemiological gradients of infectious diseases, such as those from highly active tuberculosis burden countries, for instance, will continue to be a focus for researchers. Migrants may also have a higher prevalence of certain infections due to lack of vaccination or presence of causative factors in the country of origin such as tuberculosis, hepatitis, measles, rubella, typhoid, malaria, HIV/AIDS and cutaneous Leishmania [92, 95, 102–105].

Publications relating to maternal and reproductive health constituted was approximately 13% despite that women and girls constitute approximately 48.4% of international migrants [4]. Most migrant women work in settings associated with low-skilled and unregulated labour with restrictive access to healthcare. Furthermore, studies indicated that female migrant workers may face sexual abuse, slavery, violence, and lack of access to reproductive healthcare services [106–108]. Health care needs and expectations of female migrants, particularly reproductive health needs, are often overlooked [109–116]. Pregnancy for migrant women can be a difficult experience with many risks to mother and fetus [117–125]. Evidence shows that migrant mothers are more likely to suffer from pregnancy complications, including preterm delivery and postpartum depression compared to national women. Migrant women had poorer maternal health indicators, including perinatal mortality compared to native women [126–132]. Results of the network visualization maps show that migrant children are at higher risk of domestic accidents, respiratory infections, and gastrointestinal illnesses due to poor living conditions or suboptimal hygiene and accidental poisoning than non-migrant children [93, 133–135]. The above-mentioned health-related problems of migrant women and children are foreseen as a challenge to the host countries and their health care systems as they need to provide diverse health care in different languages to people with diverse backgrounds, socioeconomic conditions, and migrations circumstances. The fact that most research has been undertaken in high-income regions indicates that the issues relating to migrant women and children in mobility corridors in developing contexts remain largely unexplored.

Only 9% of retrieved literature in GMH related to NCDs. Certain migrant groups such as migrant workers may be at increased risk of ill health due to poor management of chronic diseases such as cardiovascular disease and diabetes, due to an of interruption of health care and lack of health care access due to their legal status [136]. Studies indicate a significant burden of communicable and NCDs among newly arrived migrants and refugees at immigration reception centers and temporary immigration detention centers [137–143]. Many migrant workers are also at greater risk of exposure to injuries due to the occupational environment and work settings such as construction sites.

### Most active countries, institutions, and authors

Western Europe, North America, and Australia are the most active regions in publishing documents in GMH – a reflection of the total number of migrants and refugees in these regions and/or the number of migrant health researchers. The majority of international migrants live in Europe (76 million) followed by Asia (75 million), northern America (54 million), and Africa (21 million). By country, the United States hosted the largest number (47 million) of international migrants followed by Germany (12 million), Russian Federation (12 million), and Saudi Arabia (10 million) [4]. The size and availability of budgets allocated for research as well as the national, political, and human rights agendas toward international migrants are important factors in determining the volume of research output in GMH. This could largely explain the leading role of Europe and other wealthier nations in their research output pertaining to GMH. Of the 27 specialized journals in the field of migration; the majority were based in the UK, USA, Netherlands, Canada, and Germany. The research output in GMH from Asia is relatively low given the fact that the region has the most dense international migration corridors and the largest numbers of international migrants whose country of origin is in Asia. In 2017, India was the largest country of origin of international migrants (17 million), followed by Mexico (13 million). Other countries of origin with large migrant populations include the Russian Federation (11 million), China (10 million), Bangladesh (7 million), Syrian Arab Republic (7 million) and Pakistan and Ukraine (6 million each) [2]. Our mapping showed that most literature in GMH is published from host and destination countries, and not in countries of origin. Therefore, literature in GMH represent perspectives of high-income migrant destination countries.

Limited research funding to explore the migration and health nexus, the prioritization of migration health by public health authorities within national and regional research agendas and the lack of international research networks in GMH may contribute to the poor contribution of Asian, African, and Arab countries to GMH literature. In this regard, the recently established Migration Health and Development Research Initiative (MHADRI) network may play a crucial catalytic role in advancing research collaboration at the global level, and supporting researchers within developing nations [144, 145]. The geographical distribution of publications in GMH reflects the migration flow where research output was most prominent from destination countries rather than countries of origin. In most of the cases, the countries of origin are undergoing conflict or regional wars that make research an unaffordable luxury. Furthermore, most countries of origin of international migrants belong to low- and low-middle income countries where research funding is limited. Furthermore, the countries of origin might not have enough researchers in the field of migration health or public health to shed light on these issues. Finally, countries of origin have not prioritized migration health research within national policies or towards a systematic collection of migration health data. All these points may be used as an argument to advance the field of migration health at the global level. If evidence is to guide policies and practices for migrants and refugees, then governments, donors and international organizations to invest in building capacities of the global south scholars in undertaking migration health research.

### Typology of migrants

The results of this study indicated that there is an over-representation of refugees in GMH. This is perhaps unsurprising given that refugees are more likely to have greater physical and psychological health needs considering forced migration trajectories, poor living conditions and negative health care experiences. In addition, women, children, and older people constitute a large proportion of forcibly displaced people and may be more likely to need additional health and psychosocial support [146–153]. Literature in migrant workers was very scarce (6%), despite the total number of migrant workers being seven times higher than refugees [154, 155]. Despite their economic contributions, migrant workers, and in particular those low-skilled from lower-income nations are ‘left-behind’ in global migration health research. Particular research attention needs to be focused on gender dimensions the human rights and health vulnerabilities of female migrant workers.

### Strengths and limitations

Our study is the first to assess research activity in the field of GMH. We document the dominance of mental health publications, the linear increase in publications, and the role of international collaboration. However, our study has a number of limitations: (1) Scopus database is a comprehensive and large database that includes different disciplines, but some peer-reviewed journals are not indexed in Scopus. This is particularly true for journals published from India, China, Indonesia, Sri Lanka, and other Asian and African countries. Therefore, a number of publications in GMH were missed because they were published in non-indexed journals. (2) The current study did not include grey literature which negatively affects the total number of the retrieved documents, particularly from low- and middle-income countries. (3) In ranking countries, the authors did not segregate documents based on the affiliation of the leading author. Thus, the ranking is based on the overall contribution and not the number of documents in which the country of interest is being the leader of the research. Unfortunately, this is not doable using bibliometric methods with such a huge number of retrieved documents. (4) Results obtained from Scopus reflect the nature and data present in Scopus. Therefore, if an active author has two or more Scopus profiles, his research output might be scattered and therefore his name might not appear in the active list. Same applies when the name of an institution is written in published documents using different spellings. So, the interpretation of data regarding most active authors, institutions, and countries should be confined to the results obtained from Scopus based on the strategy stated.

## Conclusion

This is the first bibliometric analysis of the peer-reviewed literature in GMH pertaining to international migrants. The findings of this paper may be useful for health authorities, funding agencies, donors and UN agencies interested in mapping research domains and identifying the gaps within the GMH research landscape. The recent Global Compact on Migrants and Refugees that seeks to set global foreign policy agendas and action plans on migration needs to account for gaps in the evidence base for advancing an evidence-informed migration and health research agenda. The heat map highlights how GMH research does not adequately reflect global migration patterns. The contribution of countries in Asia, Latin America, Africa, Middle East, and Eastern European countries was relatively low despite the significant migration flows within these regions. Research in international migration is also being published mostly from high-income destination countries with little representation from countries of origin.

The future research plan of the authors of this study and their recommendation for the scientific community who are interested in migration health are as follows. First, undertaking in-depth systematic reviews of literature by select migrant categories, by health domain (such as mental health), and by geographical demarcation (e.g. health of migrants within the South Asian region). Second, establishing research groups to investigate health conditions of female migrant workers and endorsing their human rights. Third, promoting research in migrant women’s health in general, particularly those pertaining to maternal and reproductive health. Establishing international research networks such as the Migration Health and Development Research Initiative (MHADRI) [145] is critical to provide a platform for support to researchers, especially from developing nations, to undertake research and migration data analysis in a participatory and collaborative way. Greater investments in international research collaborations and research networks should be encouraged to help prioritize research in GMH that also meaningfully engage and support capacities for research in countries of the Global South – where the largest mobility flows occur. Finally, the results of this study will form a useful baseline to be used by researchers globally.

## Additional files

Additional file 1: Research strategy and keywords used for each search query in GMH (2000–2016). (DOCX 19 kb)

Additional file 2: A scheme showing the general search strategy with number of retrieved documents in each step. (DOCX 47 kb)

## Abbreviation

GMH: Global migration health
