# Supplementary material for: Bibliometric analysis of global migration health research in peer-reviewed literature (2000–2016)
Source: BMC Public Health. 2018 Jun 20;18:777. doi: 10.1186/s12889-018-5689-x (PMC6011263; doi:10.1186/s12889-018-5689-x)
Supplement: Supplementary file 1 — Research strategy and keywords used for each search query in GMH (2000–2016). (DOCX 19 kb) [file 12889_2018_5689_MOESM1_ESM.docx]

**Additional file 1**: Research strategy and keywords used for each search query in GMH (2000 – 2016)

| **Step** | **Item** | **Revised Scopus search strategy (keywords)** |
| --- | --- | --- |
| **1** | **Migrant worker** | ((((TITLE ( *migrant* OR transient* OR *migrat* OR overseas OR "cross-border" OR non-citizen* OR non-national* OR "domestic maid*") AND TITLE ( worker OR workforce OR laborer OR labourer OR gardener OR farmworker OR "farm-worker*" OR industr* OR poultry OR agriculture OR "high skilled" OR "low-skilled" OR driver)) OR TITLE("internat* *migrant worker*" OR "foreign home care worker*" OR "foreign domestic worker*" OR "foreign domestic helper*" OR "transnational domestic worker*" OR "foreign domestic employee*" OR "overseas domestic worker*" OR "domestic migrant worker*" OR "International Labour migrants" OR "internat* illegal *migrant*" OR "Temporary migrant worker" OR "migrant health worker*" OR "frontier migrant worker" OR "Expatriate workers" OR "Inbound *migrant* worker*" OR "irregular *migrant" OR "irregular migration" OR "irregular *migrant*" OR "labour migration" OR "labor migration") AND NOT TITLE-ABS("in migration" OR "internal migrant" OR "rural to urban migrant" OR "urban to rural migrant" OR "intra-country migrant" OR "intra-regional migrant" OR "within country" OR consular OR military OR diploma* OR "international health elective*" OR cell OR signaling OR "neuronal migration" OR physics OR amplifier OR gene OR neuronal OR neural OR invest* OR "exchange market" OR electromagnet* OR pipe OR network OR memory OR glucose OR cash OR "internal migration")))) |
| **2** | **International students** | (TITLE( "international student*" OR "foreign student*") OR SRCTITLE ( "international student*" )) |
| **3** | **Refugees, Asylum seekers** | ((TITLE (refugee* OR "asylum seek*" OR "displaced person*" OR " displaced people" OR "stateless person" OR "exile" OR "uprooted person" OR "asylum process" OR "Asylum - seek*") OR TITLE(asylum AND seek*) AND NOT TITLE ( internal* OR chamber))) |
| **4** | **Trafficked victim, victims of human smuggling** | (((( TITLE ( traffick* OR smuggl* ) AND TITLE ( human OR woman OR child* OR sex OR prostitute* OR girl* OR *migrant* OR women OR female OR victim OR people OR men OR mistress)) OR TITLE("forced labour" OR "forced labor" OR "forced prostitution" OR "sexual slavery" )) AND NOT TITLE-ABS-KEY ( *cell* OR "neuronal" OR gene OR equity OR animal OR biology OR neuroscience OR PCR OR genetic OR plots))) |
| **5** | **Patient mobility across borders** | (((TITLE ( mobility OR movement OR transfer OR smuggl*) AND TITLE ( patient* OR ill OR sick) AND TITLE ( border* )) OR TITLE("patient* *migrat*"))) |
| **6** | **International migrant / immigration** | TITLE ( "international *migrant*" OR "international *migration" ) OR SRCTITLE ( "International Journal of Migration, Health and Social Care" OR "Journal of Immigrant Minority Health" OR "Refugees and Human Rights" OR "Journal of Immigrant and Refugee Studies" ) OR ( ( TITLE ( *migrant*) AND NOT TITLE-ABS-KEY ( "in-migration" OR animal* OR fish OR salmon OR ocean OR sea OR river OR forest OR *tropical OR cell* OR neuron OR bird OR plant* OR histor* OR 4g OR "mobile system" OR computer OR engineering OR chemical OR electro* OR fluid OR mechanical OR chromatog* OR soil OR carbon OR marine OR planet OR earth OR "stage migration" OR surgery OR synthetic OR chemistry OR chemi* OR chromatog* OR neural OR brain OR muscle OR joint OR dna OR rna OR cell* OR securit* OR "return migration" ) ) ) OR (TITLE(*migration) AND (ALL(*migrant* AND health) OR AFFIL(*migration OR *migrant* OR "public health" OR "global health" OR "health equity" OR "community medicine" OR "social medicine")) AND NOT TITLE-ABS-KEY ( "in-migration" OR animal* OR fish OR salmon OR ocean OR sea OR river OR forest OR *tropical OR cell* OR neuron OR bird OR plant* OR histor* OR 4g OR "mobile system" OR computer OR engineering OR chemical OR electro* OR fluid OR mechanical OR chromatog* OR soil OR carbon OR marine OR planet OR earth OR "stage migration" OR surgery OR synthetic OR chemistry OR chemi* OR chromatog* OR neural OR brain OR muscle OR joint OR dna OR rna OR cell* OR securit* OR "return migration" )) OR ( TITLE-ABS ( *migrant* AND health ) AND AFFIL ( *migration OR *migrant* OR "public health" OR "global health" OR "health equity" OR "community medicine" OR "social medicine" ) ) OR ( TITLE-ABS ( Refugee AND health ) AND AFFIL ( *migration OR *migrant* OR "public health" OR "global health" OR "health equity" OR "community medicine" OR "social medicine" ) ) |
| **7** | **Merge 1 through 6** | 1 OR 2 OR 3 OR 4 OR 5 OR 6 |
| 8 | **Add health terms** | AND TITLE-ABS-KEY ( "physical *activity" OR suicid* OR violence OR women OR child* OR torture OR physician OR nurs* OR pharmac* OR crisis OR challenge OR global* OR leptos* OR pain OR *health* OR medicine OR cancer OR infant OR birth OR hepatitis OR emergency OR cesarean OR disease OR pregnancy OR death OR illness OR mental OR maternal OR *natal OR anxiety OR infect* OR psychiat* OR psycholog* OR stress* OR bacteri* OR viral OR virus OR parasitic OR coping OR medical OR medications OR antibiotics OR *therapy* OR trauma* OR injur* OR *nutrition* OR "food *security" OR mortality OR morbidity OR heart OR diabetes OR dermatolog* OR skin OR gastro* OR diarrhea OR safety OR hiv OR unsafe OR patient OR psych* OR hospial* OR tubercul* OR malaria OR chagas OR resilience OR depressi* OR anemia OR allergy OR drug OR "substance abuse" OR hygien* OR risk OR vulnerable OR treat* OR therapeutic OR well-being OR wellbeing OR illness OR wound* OR injur* OR disease OR disability OR infect* OR symptom* OR trauma OR "mental illness" OR mental OR "mental disorder" OR disorder OR anxiety OR depression OR depressive OR fear OR guilt OR hostility OR suicide OR "Behavioral symptom" OR "Self-injurious behav*" OR "Reproductive behavior" OR "Risk taking" OR "Sexual behavior" OR "Social behavior" OR violence OR rape OR "sexually transmitted diseases" OR hiv OR pregnancy OR "abortion" OR virus OR abuse OR fever OR outbreak OR "multi-drug resistant" OR leptos* OR transmission OR epidemiolog* OR medicolegal OR "risk factors" OR "global response" OR nurs* OR risk OR oral OR challenge OR prevalence OR contracept* OR condom OR disparity OR equality OR poverty OR poor OR communication OR "promoting health" OR governance OR "health outcome" OR neighborhood OR cardiac OR cardio* OR clinic* OR *equalit* OR "health system" OR *justice OR abuse OR "minority health" OR "women* health" OR "child* health" OR "healthcare delivery" OR "access to medicine*" OR "access to health*" OR "health equity" OR "health* polic*" OR "delivery of healthcare" OR "health* service*" OR "health* system*" OR "access to medication*" OR "access to treatment" OR "reproducti* health" OR "adoles* health" OR "maternal health" OR "Health disparit*" OR "health inequalit*" OR "health* *justice" OR "health* abuse*" OR "limited access to health*" OR "women* right*" OR "limited access to medicine*" OR "human right*" OR "health polic*" OR endocrine OR disorder OR respiratory OR care ) |
| 9 | **Exclude irrelevant terms, irrelevant journals, and irrelevant subject areas.** | AND NOT TITLE-ABS ( "Turgut Ozal University" OR cell* OR accounting OR software OR "internal* displaced" OR "internal *migrant*" OR internship OR lending OR drama OR "creative expressi*" OR touris* OR neural OR signaling OR neuronal OR gene OR protein OR cd44 OR animal OR neutrophil OR "process migration" OR chemistry OR chemical OR geograph* OR geopoliti* OR management OR macrophage OR "internal migra*" OR electromigration OR equilibrium OR "anti-immig*" OR "civil war" OR polymorphism ) AND NOT SRCTITLE ( teacher OR "social inquiry" OR anthropolog* OR discourse OR politic* OR society OR sociology OR history OR "High* Education" OR english OR linguistic OR ethnic OR ethnicity OR "contemp* law" OR urbaniz* OR education OR crim* OR gender OR budd* OR "microbiology and Immunology" OR men OR "evaluation review" OR viewpoint OR "European Journal of Women's Studies" OR bridges OR "community development" OR "driven from home" OR "black girls" OR "religious dynamics" OR "collegium" OR language OR "infant observation" OR touris* OR obesity OR "academic medicine" OR "International Journal of Educational Management" OR physics OR *econom* OR sociolog* OR surgery OR text* OR synthetic OR chemistry OR chemi* OR chromatog* OR climat* OR "journal of mammology" OR "Ecological Modelling" OR "Urban Studies" OR "Journal of Business Research" OR "land use policy" OR "Foreign Literature Studies" OR "Indiana Law Journal" OR bilig ) AND ( LIMIT-TO ( SRCTYPE,"j " ) ) |
| 10 | **Limit to study period from 2000 - 2016** | AND ( EXCLUDE ( SUBJAREA,"ENGI " ) OR EXCLUDE ( SUBJAREA," CENG " ) OR EXCLUDE ( SUBJAREA," CHEM " ) OR EXCLUDE ( SUBJAREA," EART " ) OR EXCLUDE ( SUBJAREA," ENER " ) ) AND ( EXCLUDE ( DOCTYPE,"er " ) OR EXCLUDE ( DOCTYPE," bk " ) ) AND ( LIMIT-TO ( PUBYEAR,2016 ) OR LIMIT-TO ( PUBYEAR, 2015 ) OR LIMIT-TO ( PUBYEAR, 2014 ) OR LIMIT-TO ( PUBYEAR, 2013 ) OR LIMIT-TO ( PUBYEAR, 2012 ) OR LIMIT-TO ( PUBYEAR, 2011 ) OR LIMIT-TO ( PUBYEAR, 2010 ) OR LIMIT-TO ( PUBYEAR, 2009 ) OR LIMIT-TO ( PUBYEAR, 2008 ) OR LIMIT-TO ( PUBYEAR, 2007 ) OR LIMIT-TO ( PUBYEAR, 2006 ) OR LIMIT-TO ( PUBYEAR, 2005 ) OR LIMIT-TO ( PUBYEAR, 2004 ) OR LIMIT-TO ( PUBYEAR, 2003 ) OR LIMIT-TO ( PUBYEAR, 2002 ) OR LIMIT-TO ( PUBYEAR, 2001 ) OR LIMIT-TO ( PUBYEAR, 2000 ) ) |
