# Supplementary material for: Bibliometric analysis of global migration health research in peer-reviewed literature (2000–2016)
Source: BMC Public Health. 2018 Jun 20;18:777. doi: 10.1186/s12889-018-5689-x (PMC6011263; doi:10.1186/s12889-018-5689-x)
Supplement: Supplementary file 2 — A scheme showing the general search strategy with number of retrieved documents in each step. (DOCX 47 kb) [file 12889_2018_5689_MOESM2_ESM.docx]

**Migrant workers**

**= 5002**

**International students = 2628**

**Refugees and asylum seekers = 15803**

**Human trafficking and smuggling = 1944**

**Patient mobility across borders = 58**

**International migrants**

**= 40573**

**Limit to documents in health-related field = 23777**

**Limit to journal documents = 29991**

**Limit to study period 2000 – 2016 = 32003**

**Total = 61644**

Exclude irrelevant documents (e.g. internal migration); **Net Total = 21457**

**Additional file 2:** A scheme showing the general search strategy with number of retrieved documents in each step.
